# Gluten-Free Bread and Bakery Products Technology

**DOI:** 10.3390/foods11030480

**Published:** 2022-02-07

**Authors:** Zuzana Šmídová, Jana Rysová

**Affiliations:** Food Research Institute Prague, Radiová 7, 102 00 Prague, Czech Republic; jana.rysova@vupp.cz

**Keywords:** gluten-free products, bread, bakery products, cereals, enzymes, sourdough

## Abstract

Gluten, a protein fraction from wheat, rye, barley, oats, their hybrids and derivatives, is very important in baking technology. The number of people suffering from gluten intolerance is growing worldwide, and at the same time, the need for foods suitable for a gluten-free diet is increasing. Bread and bakery products are an essential part of the daily diet. Therefore, new naturally gluten-free baking ingredients and new methods of processing traditional ingredients are sought. The study discusses the use of additives to replace gluten and ensure the stability and elasticity of the dough, to improve the nutritional quality and sensory properties of gluten-free bread. The current task is to extend the shelf life of gluten-free bread and bakery products and thus extend the possibility of its distribution in a fresh state. This work is also focused on various technological possibilities of gluten-free bread and the preparation of bakery products.

## 1. Introduction

A gluten-free diet is the only treatment for people suffering from gluten intolerance. Gluten consumption leads to a range of gluten-related disorders, such as coeliac disease, dermatitis herpetiformis (cutaneous manifestation of coeliac disease), gluten ataxia and non-coeliac gluten sensitivity [1]. A gluten-free diet requires the use of gluten-free cereals—corn, rice, sorghum, millet, teff—and pseudo-cereals—buckwheat, quinoa, amaranth, canihua—but also other foods that are naturally gluten-free—potatoes, tapioca, nuts, oilseeds, legumes, fruits and vegetables [2]. The main challenges for food technologists are bread, bakery products, pastry and pasta. Because of the absence of gluten, other substances needed to maintain the texture, volume, satisfactory crumb, shelf life and sensory quality must be used. These include the use of hydrocolloids, sourdough or enzyme preparations. The use of them is intended to change the recipe and the production technology.

Gluten-free bread and other gluten-free bakery products are very unusual for a consumer accustomed to classic wheat or wheat–rye bread. Toth et al. (2020) have shown that 70.8% of the asked consumers were dissatisfied with gluten-free breads due their texture and taste [3]. Gluten-free breads usually have a less flexible crumb, which hardens faster, and which is easy to crumble. The taste of these products is also different, depending, of course, on the ingredients used. Gluten-free products are easily accepted by people who have been suffering from gluten intolerance since childhood. The acceptance of a gluten-free diet, and at the same time, the acceptance of gluten-free bread in adults diagnosed with gluten intolerance later in life, is more difficult.

According to Codex Standard 118-1979 [4], gluten represents a protein fraction from wheat, rye, barley, oats or their hybrids and derivatives that some people are intolerant to and that is insoluble in water and 0.5 M sodium chloride solution. Water-insoluble prolamins and glutelins (collectively referred to as gluten) usually make up 70–80% of cereal grain proteins. They are the most important cereal proteins from a technological point of view. In this sense, gluten is a specific structure, a viscoelastic gel that gives wheat dough and bakery products its unique properties. This gel is formed after the addition of water and kneading, when the wheat proteins gliadins and glutenins swell, and with the simultaneous access of oxygen as a complex, a three-dimensional viscoelastic system (gluten in the original technological sense) is created which ensures the required viscoelastic properties of the dough. The result is a three-dimensional sufficiently strong and flexible continuous network capable of maintaining a large volume of gas, and thus ensuring the sufficient volume, shape and texture of the products.

**Figure 1:**
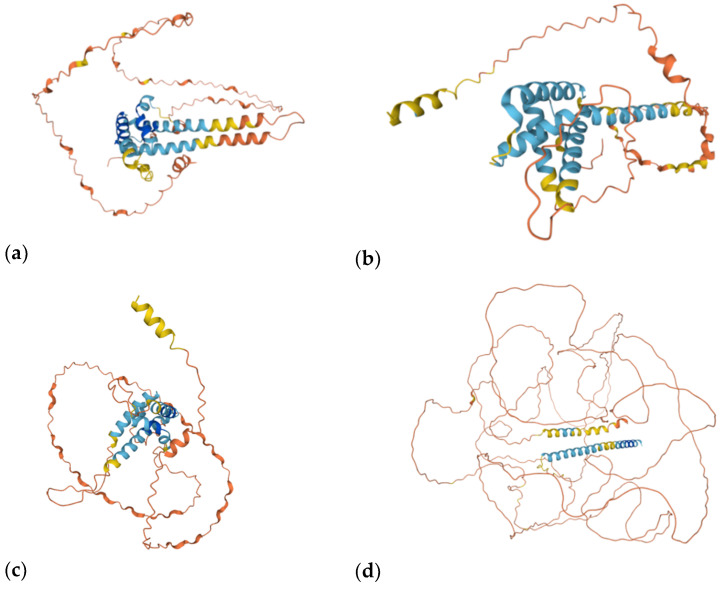
Gluten structure from the UNIPROT database https://www.uniprot.org/ (accessed date 31 January 2022) [6]. The picture shows (**a**) glutenin LMW subunit; (**b**) α,β-gliadin; (**c**) γ-gliadin; (**d**) glutenin HMW subunit. AlphaFold produces a per-residue confidence score (pLDDT) between 0 and 100. Some regions with low pLDDT may be unstructured in isolation. The colours represent the model confidence: dark blue–Very high (pLDDT > 90); light blue–Confident (90 > pLDDT > 70); yellow –Low (70 > pLDDT > 50); red–Very low (pLDDT < 50).

**Figure 2:**
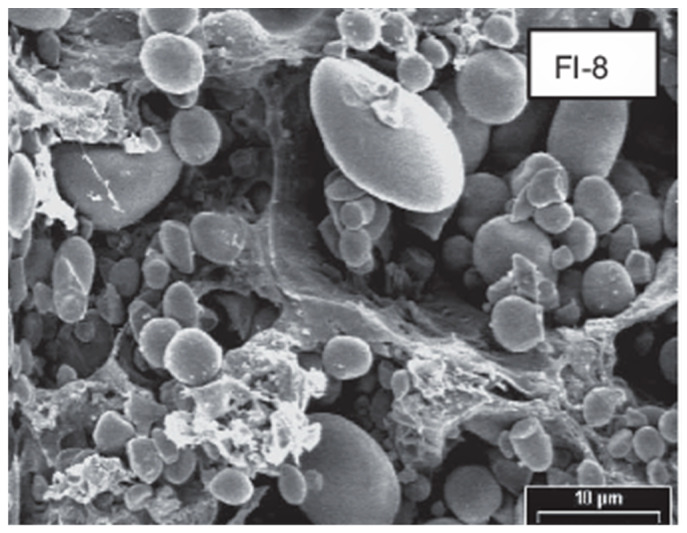
Scanning electron micrograph of the gluten dough prepared in Brabender Farinograph from a wheat flour after 8 min. of mixing (FI-8, magnification: 2600×). Adapted from [7].

Prolamins and glutelins are represented by a number of related proteins with somewhat different amino acid composition and structure (e.g., gliadin proteins are usually up to several dozen for each wheat variety). In particular, the prolamins of wheat (as well as spelt, Khorasan, einkorn and emmer), rye and barley and their hybrids (Triticale, Tritordeum) cause a disease in predisposed individuals, which is called celiac disease. The relationship between oat prolamins and celiac disease is still the subject of debate. Wheat gliadin fractions are divided into four subfractions: α-, β-, γ- and ω-gliadins (Figure 1 and Figure 2). All subfractions of α-/β-/γ-/ω—gliadins can cause celiac disease in predisposed individuals, and ω-5 gliadins allergic reaction. Celiac disease is caused by two amino acid sequences: ProSer-Gln-Gln (PSQQ) and Gln-Gln-Gln-Pro (QQQP) [5].

## 2. Raw Materials for Gluten-Free Bread and Bakery Products

The specific technological properties of typical gluten cereals in the production of gluten-free bread and bakery products need to be replaced. Rice, corn, or sorghum and other gluten-free cereals are the basis of the diet in many countries around the world. For the preparation of bread and bakery products from gluten-free raw materials, it is necessary to ensure the volume and cohesion of the dough. Rapid staling of these products is a big problem. The nutritional value of wheat and gluten-free bread can be different. Table 1 shows the comparison of nutritional values between wheat flour and gluten-free bread baking mixtures (or flour, respectively) and Table 2 the composition of fresh gluten-containing and gluten-free buns. The nutritional values strong depend on the raw material composition of these products and are not uniform.

In addition to basic gluten-free ingredients such as gluten-free flours and starches, technologically and nutritionally functional ingredients such as hydrocolloids of cereal and non-cereal origin [8], fruit or vegetable fiber [9,10], flax and chia seeds [11,12], psyllium [13], modified starches (e.g., [14]) and proteins [15] from many sources need to be added to achieve sufficient bread volume, crumb softness and shelf life. The addition of fiber, through its hydration, affects the quality of the bread. Besides the beneficial health effects, fiber improves texture, specific volume, apparent viscosity, consistency, texture, sensory quality and shelf life. This is due to the ability to bind water, form a gel and thicken [8]. The key parameters are fiber length, polymerization degree, soluble/insoluble fiber ratio and fiber interactions with other ingredients [16,17,18,19]. Soluble fiber improves dough; coarse fiber reduces gas retention [13,20]. The use of enzyme preparations improves the colour of crumbs, supports the production of flavors, increases specific volume and prevents starch retrogradation [21]. Krishna et al. (2019) [11] evaluated the effect of 1% (total base) addition of flax seed powders from flax (*Linum usitatissimum*) and acacia seed powder on the pasting properties, texture and volume of gluten-free bread. The addition of all seed powders reduced crumb hardness by 30–65% and increased specific loaf volume by 50%. These textural improvements were caused by water absorption capacity and emulsifying ability. A darker bread crumb was observed after flax addition, whereas after acacia addition, dark particles were visible. Scanning electron microscopy of these breads showed the absence of holes in the pore surface and viscoelastic starch–protein network. Steffolani et al. (2004) [12] observed a reduction in specific volume and an increase in bread hardness after the addition of chia seed or flour into rice breads, when the effect was more evident with the flour than with seeds. Chia addition minimized weight loss during baking. Chia flour addition led to a darker crust and crumb. No significant differences between the different breads in acceptability were noted; however, chia seed breads presented better texture than controls. Additionally, Sandri et al. (2017) [22] prepared chia-containing rice breads with acceptable sensory properties when the best formulations were prepared from rice flour blends with 5, 10 and 14% whole chia flour. The overall acceptability scores were 8.7, 8.1 and 7.9 out of the 10-point scale, and were very similar to their white gluten-free bread and wheat bread counterparts. The addition of chia flour was acceptable up to 14%. The use of 5–14% whole chia flour increased the levels of lipids, proteins and dietary fiber compared with the white gluten-free breads.

Moreover, there is a trend to use fermentation processes in bread baking so that the products resemble sensory properties of sourdough bread, e.g., the use of sourdough made from gluten-free flour (e.g., [23]). In addition to organic acids, the lactic bacteria also produce free amino acids as precursors of flavours, contemporary degrade phytates and starch and change the fiber solubility. The resulting organic acids and antimicrobial substances extend the shelf life of the bread. Some lactic acid bacteria produce exopolysaccharides, which can affect the rheological properties of the dough [24].

Another possibility of raw material modification is the use of controlled germination. Germinated seeds are characterized by improved taste and nutritional properties. Germination activates seed enzymes, the partial degradation of storage substances into simpler sugars or peptides occurs and the availability of minerals increases. Similar to fermentation, germination also produces a number of secondary metabolites and leads to the degradation of antinutritive substances [25,26,27].

An integral part of gluten-free dough is emulsifiers, which enable easier processing of the dough and soften the crumb. Lecithin, mono- and diglycerides of fatty acids and esters of fatty acids with lactic acid are used for this purpose. Milk, egg yolk (except for bread), soy protein, sunflower and lupine flour can also serve as emulsifiers [28,29].

The basic raw materials for gluten-free bread and bakery production are gluten-free flours or native starches to which additional ingredients must be added that “substitute gluten” and ensure optimal dough properties. Flours represent more complex materials in comparison with starches, which also include proteins and a low amount of lipids, as well as some minor components such as fiber, vitamins and minerals. Therefore, they are more convenient. For gluten-free products, there is only scarce information concerning flour requirements. These flours differ in starch characteristic (e.g., amylose and amylopectin ratio), in protein amount, and in particle size and their distribution. As far as gluten-free wheat starch present on the market is concerned, it does not have harmful effects on most celiacs; however, celiac people are still unwilling to consume products with wheat-based ingredients [30].

## 3. Gluten-Free Dough Specifications

Gluten-free dough is a very complex semi-liquid system that contains polysaccharides and other structure-forming components, viscosity-increasing substances and dough-stabilizing substances. It is characterized by high density and low elasticity. Gluten-free dough contains more water than conventional wheat dough. The amount of water depends on the nature of the basic raw materials, their ability to absorb water and the granulation of the raw materials. Additionally, the kneading, its length and its speed are very important. Prolonged kneading increases the specific volume of bread [31].

When baking, the proteins are denatured with increasing temperature, and the starch gelatinization occurs. A sufficiently strong and flexible spatial structure should be created to maintain the expanding gas bubbles and not collapse during baking or cooling of the product. However, gluten-free flours and starches alone do not create such a structure. Therefore, hydrocolloid addition is necessary because of their swelling and water binding capacity [8,32]. Proper hydration affects the conformation of polymer molecules and the rheological properties of the dough. It also determines the texture and softness of the crumb and the crunchiness of the crust. The less hydrated dough provides a small volume of bread, the more hydrated dough can be processed better and the fermentation takes place better in it. Without the addition of other raw materials, the product is irregular in shape, not very cohesive and the crumb is not sufficiently supple and flexible. If the gluten-free recipe contains less protein, the product has usually light crust. There are not enough amino acids available to enter the Maillard reaction [33,34].

After baking, the crust on the surface is firm and crunchy, whereas the crumb retains moisture. After a certain storage time, the moisture in both parts of the bakery product begins to equalize. The water retained in the crumb diffuses to the surface of the product; the crust softens and may deform. On the contrary, through this redistribution process the crumb loses water, thus reducing the flexibility and suppleness of the bread (Table 3). Starch tends to return from an amorphous state to a crystalline form. Starch is recrystallized (retrograded) and bread is staling. The crumb is now brittle and incoherent or hard [35]. Rapid staling and thus limited shelf life are a big problem with gluten-free products. Short shelf life limits the possibility of the sale of fresh gluten-free bread and is one of the reasons for using dry mixtures for home baking of gluten-free bread and bakery products [33].

The viscoelastic properties of different doughs significantly influence the volume, and the crumb texture of gluten-free baked goods [41]. One of the most important factors affecting the quality of bread and bakery products is the flour particle size. For bread production, bigger particle size is more suitable and particles below 80–100 µm should not be used if gluten-free bread with high volume and soft crumb is desired. Flours with very large particles may result in breads with sandy texture; therefore, 200 µm is the maximum particle size. Flours with larger particles have been proven to reduce the dough gas retention capacity as well as the final bread volume [30].

### 3.1. Proteins in Gluten-Free Dough and Products

Proteins improve the nutritional value of gluten-free products. The choice of flour and possibly another source of protein affects the rheological properties of the dough and the water binding in the dough. Proteins interact with starch and lipids and together contribute to the stability of the dough and the structure of the product. They also give the impression of full product flavour. Proteins can be of plant origin (legumes, soya, gluten-free cereals, rapeseed, canola, sunflower, potato), animal origin (whey, egg, casein, caseinate) or microorganism-, algae-, seaweed- and insect-based [15].

Conventional proteins represent egg and milk proteins. Eggs are very useful in forming the structural network, but they are not usually used in bread. Milk proteins, including caseinates and whey protein concentrates, are sources of calcium and can bind moisture satisfactorily. They have a positive effect on the colour and volume of bread and bakery products. For example, protein-rich gluten-free bread with the addition of 15% whey protein concentrate and 3% of HPMC were prepared by Rustagi et al. (2018) [42]. However, many celiacs do not tolerate lactose and must omit milk from their diets [43].

**Figure 3:**
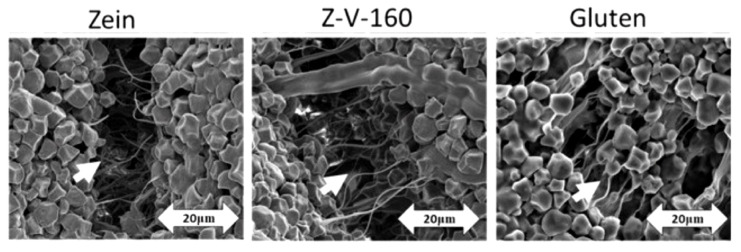
Images of dough samples containing starch with zein, thermally treated-zein (zein heated in vacuum at 160 °C, Z-V-160) and gluten. Adapted from [46]. The white arrows in the image represent the scale-they indicate the distance of 20 µm.

The source of proteins are naturally gluten-free cereals—rice, corn, teff, sorghum, Job’s tears (Figure 3). These cereals contain prolamins too (an ethanol-soluble protein fraction), but the molecules of these proteins do not contain amino acid sequences that are toxic to people with celiac disease. Rice is often used as the basis for gluten-free bread formulations. Gluten-free oats have also very good properties [44]. The zein protein, a prolamin from corn, behaves similar to gluten when heated to 35–40 °C. Both corn zein and sorghum kafirin increase the plasticity of the dough [31,45].

Cereal flours are combined in gluten-free bread and bakery products formulations with flour from other crops and with starches. A combination of cereals with legumes is nutritionally advantageous. Pseudocereals also contain proteins with a preferred amino acid composition [8]. This category includes buckwheat, amaranth and quinoa. For example, Föste et al. (2013) [47] used various buckwheat milling fractions, rice and corn flour and fermented buckwheat brans for gluten-free bread preparation. The specific bread volume, porosity and crumb texture can be improved by using buckwheat flour. Soya is a traditional gluten-free ingredient. Soy protein has a very advantageous amino acid composition, participates in interactions with other substances, binds water well and slows down the staling of bread. Legume flours from pea, chickpea, lupine, lentil and bean are rich in proteins with a high lysine content. They significantly affect the dough quality. Lupine and soya flour show emulsifying properties because of the lecithin content. Legumes reduce the glycemic index of food products. The disadvantage of legumes is their typical taste [35,48,49,50]. Recently, other flours, such as from nuts and seeds, have appeared in the range of bakery products. Typically, walnut flour and peanut flour are relatively expensive, but very suitable for some formulations. Fat in walnut flour or other nuts flours (except for coconut) contains polyunsaturated fatty acids, and nut proteins are also of very high quality due to their composition. Coconut flour binds water very well, too [51,52].

An alternative solution is the use of insect proteins, e.g., cricket flour which improves the texture of gluten-free bread [31].

The use of proteins in the form of protein concentrates or isolates from different sources in gluten-free baked goods leads to the quality and nutritional profile improvement [15]. Through the comparison of plant- and animal-based proteins, Gorissen et al. [53] reported lower content of essential amino acids in plant-based protein isolates than in animal-based proteins. Differences in the composition of the amino acid spectrum of gluten-free raw materials are known, especially in the composition of essential amino acids. Cereal proteins are deficient in lysine, and some cereals also in threonine and tryptophan. Therefore, from this point of view, it is recommended to combine different types of plant proteins and thus optimize the ratio of amino acids. The combination of different ingredients makes easier to ensure the presence of other nutrients, such as vitamins or minerals.

### 3.2. Starch in Gluten-Free Dough and Products

Starch, together with flours from gluten-free crops, is one of basic ingredients in gluten-free bread and bakery products. It is involved in the formation of the crumb structure, responsible for the volume and colour of the product. It is also used as a thickening, gelling, stabilizing, moisture retention and anti-staling agent [54]. According to Abdel-Aal (2009) [55], starch influences gluten-free products in three ways: it enhances crumb softness, ensures dough consistency and affects starch gelatinization. Starch is stored in starch grains of various sizes and shapes according to its plant source. Individual starches differ in their composition, size and shape depending on the plant species and the interactions between genes and environment [54]. The amylose starch fraction forms single chains, whereas amylopectin is branched with a significantly larger molecule. When heated in suspension/dough, the starch grains swell, are partially solubilized [56], and gradually lose their cohesiveness. Starch gelatinization occurs at a temperature of 50–70 ° C, when their chains are released, and a viscous solution is formed from the suspension. Upon cooling, the viscosity increases, new bonds are formed between the molecules and a gel is formed. During storage, the gel further changes, loses water and eventually retrogrades. Amylose retrogradation proceeds faster than the same process for amylopectin. It follows that by choosing the type of starch, it is possible to partially influence the staling of the bread [54]. Starch behavior may be affected by bound lipids.

Native starches are the most commonly used in gluten-free products, e.g., potato, corn, rice and tapioca, and pea starch has also appeared. Specially prepared gluten-free wheat starch is also used for its properties ensuring an optimal bread texture [15,57]. Modified starches are produced for food purposes have a wide range of physical properties according to the purpose they are used. Starches can be modified by heating of the starch solution or by heating in the dry state; the heating can be performed by drying or extrusion. Chemical modification of starch is also possible [58]. For gluten-free products, starches with good water absorption and slow retrogradation are selected. Specially modified starches are suitable for frozen products. A new modification is the so-called superheated starch, prepared by heating the starch suspension to high temperatures until dissolved and then cooling to form a spreadable gel with a creamy consistency [59,60]. Additionally, various types of banana flour can be applied as well as the direct use of bananas in the dough [61].

There are significant differences in granular structure among various types of starches, which affect their ability to produce high quality gluten-free baked goods. When the baked goods are based on starch, they show higher volume, lower hardness and a lighter crust since Maillard reactions are reduced. The starch addition results in softer, and resilient crumbs. The type of starch also influences the quality of the baked goods. For the specific gluten-free formulations different mixtures of flours and starches must be optimized [30,41].

Preventing the retrogradation of starch and thus prolonging the shelf life of gluten-free bread and bakery products can be achieved in several ways:

(a) Using enzyme preparations.
(b) Application of hydrocolloids.
(c) Using sourdough fermentation.
(d) Suitable packaging method.

#### 3.2.1. Use of Enzyme Preparations

The most common enzyme preparations use amylases, which improve the colour of crumbs and support the production of flavors. Amylases partially degrade amylopectin and thus modify the starch recrystallization process [62,63,64]. Transglutaminase improves the dough viscoelasticity and decrease crumb hardness, and cyclodextrinase also enhances dough viscoelasticity, leading to improvement in shape index and crumb firmness [65].

Cyclodextrin glycosyltransferases form cyclic structures from starch with different affinities for water outside and inside the ring. Lactase and tyrosinase create crosslinks of non-starch polysaccharides with proteins with the use of phenolic substances [25]. Oxidases such as lipoxygenase, sulfhydryl oxidase, glucose oxidase and peroxidase stabilize the dough; for example, glucose oxidase added to rice bread improved volume and reduced stiffness. Proteases and peptidases improve the interaction between protein molecules and starch and reduce the viscosity of the dough [31,66]. Microbial transglutaminase, which forms covalent bonds between the free epsilon-amino group of lysine and the amide group of glutamine, is used to promote the formation of a spatial network of gas bubble trapping molecules. Transglutaminase supports the rheological and viscoelastic properties of the dough [67,68,69,70,71]. Silva et al. (2020) [72] tested gluten-free bread from red rice flour and cassava flour, with the addition of transglutaminase and chitosan at concentrations of 0%, 1% and 2%. Bread with chitosan and transglutaminase showed lighter brown coloration because of incomplete Maillard reaction and low specific volumes, probably related to chitosan interference with yeast fermentation. With the use of chitosan, viscosity increased. Bread containing chitosan had a lower rate of staling due to water retention.

The use of enzymes influences the quality of the gluten-free baked goods, and the effect depends on the type of the flour used. Some enzymes have positive effects on product volume and delay staling [30].

#### 3.2.2. Use of Hydrocolloids

The application of hydrocolloids is crucial for the quality of gluten-free bread. Hydrocolloids swell and form a gel. This heated gel thickens the mass of dough forming the walls of gas bubbles, preventing the loss of gas released during whipping, leavening or from raising agents. After baking, hydrocolloids stabilize the crumb structure, bind water, and prevent rapid starch retrogradation. They stabilize the product during freezing. Due to the higher water binding, the recipes with the addition of hydrocolloids contain higher doses of water [32,73].

Vegetable gums (guar gum, locust bean gum, arabic gum, tara gum, carob, konjac gum), beta-glucans, pentosans and arabinoxylans, cellulose derivatives (methyl cellulose MC, carboxymethyl cellulose CMC, hydroxypropylmethyl cellulose HPMC), microbial exopolysaccharides (xanthan, dextrins) and seaweed polysaccharides (agar, carrageenans) are used to produce gluten-free bread and bakery products [42,74,75,76]. Flax flour or ground chia seeds are sometimes used to increase viscosity.

Cellulose derivatives, especially CMC and HPMC, are among the most used hydrocolloids in gluten-free dough. They can interact with other raw materials in the matrix. They are most often combined with other types of thickeners, proteins, and emulsifiers [66]. For example, Liu et al. (2018) [77] compared the effect of HPMC, CMC, xanthan gum and pectin on the behavior of steamed potato dough. The addition of 2% HPMC increased mostly the specific volume of bread and porosity and reduced the stiffness of the crumb by almost 29%. The addition of hydrocolloids significantly reduced the content of both readily available and slowly available starch and, conversely, increased the content of resistant starch. Model experiments with HPMC and rice flour on Mixolab examined the effect of HPMC dose (1–3%) and water dose (90–110%) on the rheological properties of gluten-free dough and crumb quality. The optimal dose is 2.2% HPMC and 110% water) [78]. Lazaridou et al. (2007) [79] showed that 1% CMC and 2% pectin led to breads with improved breads volume, porosity and crumb elasticity. With the use of HPMC, Hager et al. (2013) [80] observed an increased volume in corn and teff breads, a decreased size of rice breads and a positive effect on the crumb hardness of each bread.

Salehi (2019) [41] dealt with the application of HPMC, CMC and other hydrocolloids in rice flour dough. HPMC in combination with carrageenan forms a softer crumb. To slow down the aging of bread, the addition of CMC or HPMC 0.1–0.5% is recommended. Kaur and Chopra (2018) [61] tested 74% corn starch bread with tapioca, rice flour and 2.2% HPMC. Belorio and Gómez (2020) [81] tested the use of different types of hydrocolloids (HPMC, xanthan and psyllium) in rice and corn bread and the effect of water levels. The water dose has been optimized to form a thermoreversible gel that provides a sufficient volume of bread. For corn bread with added HPMC, the optimal hydration was 80%; for rice bread with HPMC, the hydration was higher—100%. 

Fruit and vegetable pomace containing fiber and antioxidants can also be used [9,41,82]. Djeghim et al. (2021) [9] observed the addition of various by-products with gluten-free bread formulations based on corn and chickpea flours (2/1 *w*/*w*)—orange and apple pomace, tomato peel, pepper peel, prickly pear peel and prickly pear seed peel on the dough rheology and properties of gluten-free breads. They found out that the addition of the above-mentioned by-products significantly improved the specific volume of gluten-free bread, with values increasing from 1.48 to 2.50 cm^3^/g, and increased the maximum dough height, the total CO_2_ production and CO_2_ retention coefficient.

The effect of apple, orange and carrot pomace powders, on dough rheology and quality characteristics of the rice sweet bakery were studied by Kirbas et al. (2019) [10]. With an increase in the content of pomace powders, the dough elasticity, specific gravity and apparent viscosity increased. The addition of pomace powder increased crumb hardness and decreased the specific volume of the rice-based sweet bakery products. The addition of 5% of orange pomace powder had the highest acceptance scores with respect to the colour, flavour, texture, appearance and acceptability of the products.

The comparison of breads and bakery products prepared from gluten-free flour alone and with various hydrocolloids (gums) showed that the incorporation of hydrocolloids led to a significant improvement in the texture, volume, color, appearance, flavor and overall acceptability. Different hydrocolloids have slightly different effects on rheology, texture and other properties, thus affecting the resulting quality of various types of gluten-free breads and bakery products. For example, xanthan gum is able to maintain unchanged texture parameters during storage; the addition of xanthan, carrageenan and guar gums decrease dough extensibility, whereas arabic gum and HPMC lead to increased extensibility [41]. HPMC is preferred to other hydrocolloids since it provides gluten-free products with appropriate physical characteristics, higher specific volumes of products and better sensory properties [30]. Moreover, the use of hydrocolloids is the easiest way to raise the content of dietary fiber in gluten-free baked goods [8].

#### 3.2.3. Microbial Fermentation in Gluten-Free Bread Production

The use of sourdough is a traditional procedure in conventional baking technology. In the preparation of gluten-free bread, starter cultures began to be applied later, because gluten-free raw materials have a specific composition different from rye flour; therefore, the classical culture of rye sourdough bacteria and yeasts may not grow sufficiently in gluten-free substrates. Although it is possible to gradually “dilute” rye flour with gluten-free raw material during repeated fermentation so that the proportion of rye is reduced to a minimum value, such a process would take a long time, and there would still be the danger of the presence of gluten traces [83]. Therefore, suitable strains of microorganisms capable of fermenting rice, buckwheat, sorghum or corn flour are sought. The choice of a suitable starting culture will significantly affect the resulting properties of the dough and the product. Additionally, the oilseed, chia and flaxseed sourdoughs can be used [84]. During fermentation, the dough is acidified. At the same time, the enzymes naturally contained in the flour are also activated and break down high molecular weight substances, and thus make them more accessible. The activity of the cereal grain’s own enzymes is combined with the action of microbial enzymes. Substances affecting the taste and smell of the products are formed. Fermentation increases the swelling of carbohydrates and improves the viscoelastic properties of the dough. The fermentation products include organic acids with a predominance of lactic and acetic acids, but some strains also produce propionic acid [85,86,87,88,89]. These acids significantly increase the shelf life of the bakery products. For example, Kaur and Chopra (2018) [61] deal with the use of teff flour and rice sourdough as a possible combination. Bacterial strains of sourdough microflora, for example *Lactobacillus reuterii* or *Weisella cibaria*, are able to produce the exopolysaccharides fructan, levan, dextran or reuteran. These polysaccharides naturally increase the viscosity of the dough and thus contribute to the formation of the product texture. The presence of these polysaccharides reduces the hardness of the crumb, improves its porosity, and slows down the staling of the bread [25,31]. Additions of dried sourdough are also being applied. The advantage of dried sourdough is its standardized quality, the disadvantage is the possible inactivation of living microbial strains during the drying process [90]. A non-traditional sourdough using *Lactobacillus sanfranciscensis* for fermentation of chia, quinoa and hemp flour to produce gluten-free corn/rice bread was tested by Jagelaviciute and Cizeikiene (2021) [87]. This sourdough showed a decreased pH, specific volume and rate of bread staling and, on the other hand, increased bread porosity compared with bread made only with chia, quinoa or hemp seed flour. The use of non-fermented chia and hemp flour increased the firmness and the rate of bread staling, whereas use of non-traditional hemp and quinoa sourdough reduced the rate of bread staling.

The use of sourdough in gluten-free baked goods leads to products with improved technological and nutritional properties [23], which are softer, tend to stale more slowly and have a delayed mould spoilage rate and thus a prolonged shelf life. Sourdough also brings nutritional benefits because it makes minerals more available and its presence leads to the production of exopolysaccharides, which function as hydrocolloids [30].

### 3.3. Gluten-Free Bread and Bakery Products Spoilage

Because gluten-free bread contains more water, it has a higher water activity. It is not usually baked using sourdough; therefore, the possibility of infestation by mould and other microorganisms is a significant problem. Mould species involved in bread and bakery products spoilage have been identified; they are represented by fungi of the genus *Penicillium*, *Cladosporiun*, *Neurospora or Rhizopus*, *Aspergillus*, *Fusarium*, *Mucor*, *Endomyces*, *Chrysonilia*, etc. [91,92]. Mould contamination leads to off-flavour generation and mycotoxins production, endangers human health and causes economic losses and consumer dissatisfaction [93]. Baked goods can also be attacked by yeasts, e.g., of the genera *Pichia*, *Candida* or *Torulaspora* and bacteria of the genus *Bacillus* (e.g., *B. subtilis*, *B. amyloliquefaciens*, *B. licheniformis*, *B. cereus*), with *B. amyloliquefaciens* as the main species causing rope spoilage [91,94]. The same microorganisms cause spoilage of gluten-free products [91].

To ensure the shelf life of bread and bakery products, various physical methods can be applied. Ultraviolet light, infrared treatment, microwave heating and ultra-high-pressure treatments can be used for bakery products preservation [95]. The disadvantages include low penetration ability of ultraviolet light, higher cost of infrared treatment or condensation problems asssociated with microwave heating [96]. The application of modified atmosphere or gamma irradiation alone does not give advantageous outputs, in practice, more types of protective factors will have to be combined. The use of antimicrobial compounds extracted from plants—biopreservation—provides very promising results and is considerably efficient in slowing down the growth of fungi [93].

**Figure 4:**
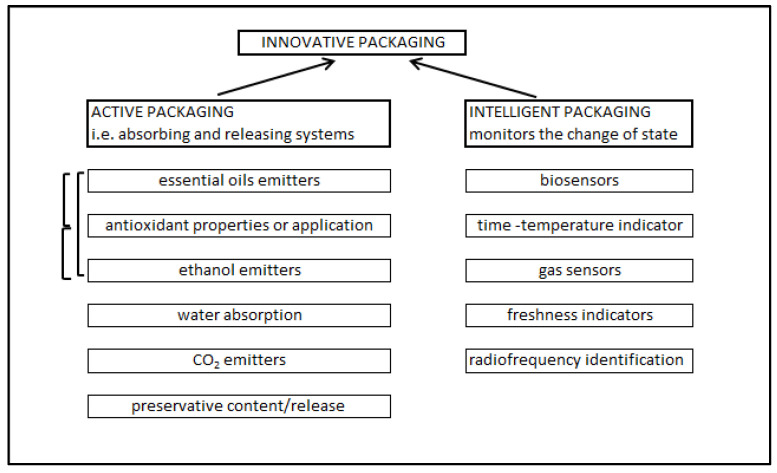
Innovative food packaging systems adapted and modified from [96,101]. Synergies between particular packaging types are marked with brackets.

For the distribution of finished bread, buns and other bakery products, it is necessary to choose packaging material with good barrier properties. It is possible to directly use packaging with antimicrobial and antioxidant properties, ethanol emitting or carbon dioxide emitting packaging, moisture absorbing packaging or packaging ensuring chemical preservation of the product, for example by potassium acetate, calcium propionate or potassium sorbate (Figure 4) [96,97,98]. Barrier packaging using oxygen absorbers and the use of modified active packaging (MAP) is a way to extend the shelf life of products. Modified atmosphere packaging [99] is now commonly used for food packaging. The use of natural essential oils is also tested for wheat bread and other bakery products’ shelf life extension; the use of this treatment can be proposed in gluten-free bakery products, too [91,100,101,102].

The use of gluten-free sourdough improves the microbial stability of bread and bakery products as well as their taste [103,104] and represents another tool of biopreservation. In addition to lactic acid, sourdough bacteria also produce acetic and propionic acids with antimicrobial properties. Axel et al. (2016) [103] found out that the addition of *Lactobacillus reuteri* R29 containing sourdough extended the shelf life by 2 days for rice and quinoa bread compared with controls. Similar results were achieved with *Lactobacillus amylovorus* DSM19280-inoculated quinoa sourdough bread [104]. Some lactic acid bacteria in sourdough also form bacteriocins directed against competing microorganisms, and thus improve the shelf life of bakery products [105,106,107].

Many possibilities exist as to how to avoid spoilage in gluten-free baked goods. To prevent spoilage of these products and prolong their shelf life, it is necessary to combine several types of protective measures and thus use their synergistic effect.

### 3.4. New Technologies in Gluten-Free Dough and Bread Preparation

Recently, several technological processes have been tested to influence the properties of gluten-free dough and improve baking. Treatment of the dough with a high pressure (pascalization) of 100–1000 MPa reduces the temperature of starch gelatinization and change the properties of proteins, including crosslinking. Starch swells and gelatinizes without granules degradation; the extent of swelling depends on the intensity and length of pascalization. This changes the viscoelastic properties of the dough, increases its flexibility, but sometimes also its viscosity [25,31]. The experiments were also performed using ultrasound and micromilling to reduce flour particles. However, no positive effects on bread volume and porosity have been found [31,45].

The properties of gluten-free dough can also be influenced by heating of dry ingredients before dough preparation. Protein denaturation and partial gelatinization of the starch occurs, which increases the flexibility of the dough and the ability to retain gas. The dough viscosity, resistance and stiffness increase, as well as the dough volume [31,108]. Microwave heating was used to heat the rice flour with a moisture content of 20–30%. Proteins denatured after opening their three-dimensional structure. The specific volume and elasticity of bread has significantly improved, and the staling of bread has slowed down [109]. The “Instant controlled pressure drop” technology based on the heating of gluten-free flour for a short time under reduced pressure was tested on a mixture of rice and bean flour. The temperature was in the range of 100–165 °C, and the pressure of 5 kPa and the heating time of 20–60 s were used. The appropriate heating conditions of gluten-free raw materials were determined so that the bread baked from the heat-treated mixture resembled the control wheat bread [110].

Microwave and infrared technologies are also tested in the gluten-free bread baking process. Microwave heating would be cost effective and fast, but the resulting product had low volume, a solid crumb and was rapidly subject to the staling process. The poorer energy penetration into the bread mass is the disadvantage of infrared heating technology, but the resulting product was better sensory evaluated. The so-called “jet-impingement” using hot air convection heating on the surface of the bread was also tested. Homogeneous heat transfer occurred, but the process was energy consuming. The disadvantage was the formation of a solid crumb and dense texture, loss of water and aroma. The starch gelatinization was not complete and starch digestibility was thus decreased. The combination of both methods was recommended for this reason [111,112].

Ohmic heating was the other tested technology. The food material was heated up by its resistance during the electric current passage. The advantage of this process would be the homogeneity of the heating [66]. Another possibility of heating is the partial baking under the reduced pressure. In the first stage, bread was baked at a normal pressure at 180 °C, and after the formation of a solid crust preventing the bread collapsing, it was baked at a pressure reduced to 60 kPa. No changes in bread volume or stiffness were observed, but product moisture was lost and the crust colour was affected. After vacuum baking, other types of starch crystals were formed in the bread and the bread tended to grow stale more slowly [113].

The new technologies improving the gluten-free dough and products quality and shelf life have been still evolving. Some of them have proven to be appropriate. It has been shown that some promising methods do not give completely satisfactory results. Other technologies need to be combined. Research in this area will certainly continue. In practice, however, the economic side of the process would be crucial. Gluten-free bakery products should be not only of high quality, but also be affordable for customers.

## 4. Clean Label vs. Gluten-Free Products

Green label means that the products do not contain additives. New consumer demands are moving in this direction [114]. However, in gluten-free products, this is very difficult because of the poor baking properties of gluten-free raw materials, missing texturing properties of wheat gluten, etc. Only some of the used additives are appropriate for clean label products. A solution can be achieved using reformulations [115]. To mimic the gluten function hydrocolloids are used which are all classified as food additives and have their E numbers in accordance with EU regulation No. 1333/2008 [116]. Psyllium and beta-D-glucan can be used as gluten replacements in clean label formulations [117]. As far as enzymes are concerned, there are some exceptions they do not have E numbers.

Chia seeds, buckwheat flour and flax flour absorb water very well; lupine flour or soy flour can serve as natural emulsifiers, thus they do not have E numbers. An appropriately chosen starting culture for the preparation of sourdough improves the shelf life and taste of the bakery products, but can also modify the rheology properties of the dough due to the production of exopolysaccharides [118]. The sourdough can also be used in clean label formulations. Flours modified by heating or extrusion are tested instead of E numbers labeled thickeners [119].

## 5. Conclusions

Gluten intolerance is becoming more common in the population, and patients with this intolerance must follow a gluten-free diet. Bread and other bakery products are staple foods and pose a problem in a gluten-free diet due to their short shelf life and the need to replace gluten. Naturally gluten-free cereals and pseudocereals, but also milled legumes, seeds and nuts, are being increasingly used for the preparation of gluten-free baked goods. The additions of hydrocolloids are traditionally used in gluten-free product formulations. Recently, amylase, transglutaminase and other enzymatic preparations have been applied to bakery gluten-free mixtures. The use of fermentation for native sourdough preparation or the addition of dried sourdough to dry baking mixes improves the taste and shelf life of gluten-free bread. The quality of gluten-free dough and bread is improved by the addition of modified starches and protein isolates or concentrates. Rheological properties of gluten-free dough, the texture and sensory quality of gluten-free bread also improve the utilization of sourdough with specific microbial strains selected for the gluten-free raw materials. Newly tested baking technologies could improve the texture and slow down the staling of these products. New packaging materials and packaging methods can affect the shelf life of gluten-free bread and pastries. In the future, it is possible to anticipate the use of other non-traditionally processed gluten-free raw materials as well as new technologies for the sourdough preparation and bread baking.

## Figures and Tables

**Table 1:** Nutritional values of wheat flour and gluten-free bread baking mixtures. The values were obtained from the product packages or available on https://itesco.cz/ (accessed on 24 January 2022).

Flour	Wheat Flour	Gluten-Free 1	Gluten-Free 2	Gluten-Free 3	Gluten-Free 4	Gluten-Free 5
Nutritional Values	per 100 g	per 100 g	per 100 g	per 100 g	per 100 g	per 100 g
Energy (kJ)	1430	1517	919	1490	1497	1475
Energy (kcal)	337	362	219	356	358	351
Fats (g)	1	1.9	4.4	0.7	5.6	0.9
of which saturates (g)	0.2	0.5	1.9	0.1	0.6	0.2
Carbohydrates (g)	69	81.9	42	84	66	80
of which sugars (g)	2	3.8	<0.5	<0.5	0.8	1.4
Proteins (g)	12	3.2	2.3	2.4	7.2	2.7
Fiber (g)	2	-	1.1	-	6.0	4.4
Salt (g)	<0.005	0.2	1.4	1.5	2.5	0.83

**Table 2:** Nutritional values of wheat buns and gluten-free buns. These values were obtained from the product packages or available on https://itesco.cz/ (accessed on 24 January 2022).

Fresh Bun	Conventional	Gluten-Free
Nutritional Values	per 100 g	per 100 g
Energy (kJ)	1352	1144
Energy (kcal)	320	272
Fats (g)	5.4	8.9
of which saturates (g)	1.6	1.8
Carbohydrates (g)	55.8	42
of which sugars (g)	1.2	3.9
Proteins (g)	10.0	4.4
Fiber (g)	2.9	3.1
Salt (g)	1.5	1.3

**Table 3:** The table shows the generalized differences between wheat dough and bread and between gluten-free dough and bread. Adapted and modified according to [34,36,37,38,39,40].

	Wheat Flours	Gluten-Free Flours
Raw materials		
swelling	good	better
Dough		
repeated kneading	yes	no
stickiness	no/small	typically high
Dynamic oscillation rheometry		
G’storage modul	lower	higher
G´´loss modul	lower	higher
phase angle tg(d)	higher	lower
Extensograph		
extensibility	high	poor
extensibility resistance	high	mostly lower
R/E ratio	mostly lower	mostly higher
area under the curve (extensibility energy)	high	very low
Farinograph		
development time	low	different according to the raw material
stability	high	different according to the raw material
degree of softening	not a clear trend	not a clear trend
water binding	mostly lower	mostly higher
Bread		
volume	high	low
crust color	darker	light
crust	crunchy	more moist, dense
crumb elasticity	good	low
porosity	good	low
pore size	large	small
staling rate	slow	faster
crust moisture	optimal	more moist
crumbliness	low	significantly higher
hardness	soft	higher

## Data Availability

Not applicable.
